# Wafer defect recognition method based on multi-scale feature fusion

**DOI:** 10.3389/fnins.2023.1202985

**Published:** 2023-06-02

**Authors:** Yu Chen, Meng Zhao, Zhenyu Xu, Kaiyue Li, Jing Ji

**Affiliations:** ^1^Research Center for Applied Mechanics, School of Electro-Mechanical Engineering, Xidian University, Xi’an, China; ^2^Shaanxi Key Laboratory of Space Extreme Detection, Xi'an, China; ^3^Shenzhen Institute of Advanced Technology, Chinese Academy of Sciences, Shenzhen, Guangdong, China

**Keywords:** wafer defect, deep learning, recognition, multi-scale feature, denoise

## Abstract

Wafer defect recognition is an important process of chip manufacturing. As different process flows can lead to different defect types, the correct identification of defect patterns is important for recognizing manufacturing problems and fixing them in good time. To achieve high precision identification of wafer defects and improve the quality and production yield of wafers, this paper proposes a Multi-Feature Fusion Perceptual Network (MFFP-Net) inspired by human visual perception mechanisms. The MFFP-Net can process information at various scales and then aggregate it so that the next stage can abstract features from the different scales simultaneously. The proposed feature fusion module can obtain higher fine-grained and richer features to capture key texture details and avoid important information loss. The final experiments show that MFFP-Net achieves good generalized ability and state-of-the-art results on real-world dataset WM-811K, with an accuracy of 96.71%, this provides an effective way for the chip manufacturing industry to improve the yield rate.

## 1. Introduction

With the rapid development of technology and society, semiconductor manufacturing has become one of the most essential industries in the world (Chen et al., 2020) and wafer processing is the basis of it (Bengtsson, 1992). Due to the increasing complexity of semiconductor processes and an increase in the number of wafers produced (Chang et al., 2005), the amount of online and offline data required for diagnosis yield conditions has grown exponentially (Liao et al., 2013), with many of these wafers found to be defective on inspection. Wafer fabrication usually requires a series of processes such as photolithography, deposition, ion implantation, diffusion, machine handing, and chemical mechanical planarization (Cheon et al., 2019). Defects in wafer fabrication arise from variations in the manufacturing process, and defects in a single wafer can render the product in question completely ineffective or even discard the entire batch, so it is important to detect defects and improve the yield. But defects in wafer diagrams have a high tendency to derive necessary information about specific manufacturing process problems from different defect diagrams (Chen and Liu, 2000). Typical spatial patterns in Wafer Maps (WMs)consist of edge-ring, center, scratch, donut, and near-full, etc. (Wang et al., 2019) A center often arises due to problems in the thin film deposition, a ring is due to problems in the etching step, a scratch is a result of machine handing problems (Wang and Bensmial, 2006) and particle-type defects can be fixed by cleaning the surface with an air blower (Cheon et al., 2019). As the process issue happens, engineers can analyze the defective type of wafers to identify the root causes of the problem and reduce the loss caused by excursion (Chien et al., 2007) as soon as possible. Since all tasks of improving yield require engineers to analyze and process large amounts of data, defect pattern recognition is usually performed through statistical data analysis (Chen and Liu, 2000). Cunningham and MacKinnon (2002) divided the common visual defect metrology into three types.

1. Quadrat statistics: the defect distribution on the wafer is analyzed to predict the yield model, such as by using the conventional Poisson model and Murphy model (Berglund, 1999). Many models (Collica, 1990; Weber et al., 1995; Nurani et al., 1998; Wang et al., 2002) have been based on this type of defect metrology statistics, but this type of defect metrological method has ignored spatial pattern and defect clustering phenomena (Chen and Liu, 2000), and when the data of the wafer does not meet the hypothetical assumptions, it does not work well.
2. Cluster statistics: wafer defects are often determined by defect coordinates, when one or more wafer defects are defined, they can be classified according to the characteristics of the coordinates. This type of method seeks clusters with high defect density and ignores information about the signatures of clusters, such as the shape and size, etc.
3. Spatial pattern recognition: besides defect clusters, the spatial pattern of the defects usually provides a good approach to wafer problem solving. Ken et al. (2002) outline that special shapes appearing on the defect map pattern may come from the machine or process, according to different map patterns, then can find out the root of problems.

Accurate and efficient wafer defect detection technology can identify production process problems and make adjustments to the production process in a timely manner, thus improving the quality and yield of production wafers. To address the problem of wafer defect detection and identification, operators have traditionally visually inspected defects and classified and identified them according to predetermined methods. However, this approach involves a great deal of effort and costs being invested in pre-training defect inspection and the classification of operators (Chen and Liu, 2000). Due to the influence of human factors, the results identified by different operators are different even for the same type of defect (Weber et al., 1995). Therefore, to save costs and improve accuracy, researchers have conducted a series of studies. In the classification of technology and automatic detection of semiconductor manufacturing, frequency domain filtering using optical methods, laser irradiation scanning, and various digital image processing techniques are applied to wafer surface image detection and mostly employed by charge coupled device cameras (Qu, 2002). Most automatic inspection systems scan the wafer surface to collect the coordinates of areas where defects may exist, then place a camera at the center of the coordinates to take pictures, before automatically performing defect detection. Due to the microcosmic nature of the scanning electron microscope sensing field, it is difficult to analyze and detect the surface characteristics of the whole wafer, and the classification accuracy is poor (Cheon et al., 2019), meaning manual detection is required to measure the physical parameters of the WMs like location, size, and color later (Lee et al., 2017). Moreover, Auto Detect Camera (ADC) based approaches apply machine learning and image recognition for wafer defect classification and are introduced to reduce labor and manufacturing costs. Knights Technology (Chen and Liu, 2000) proposed a software program named spatial pattern recognition, the core of the software is a signature classifier, which can be used to train models for different batches of wafer defects, but it takes a lot of time and has poor generalization in training new models. Lee and Inc (1996) propose a templates matching algorithm to detect wafer defects, which is based on the supervised learning method, and improves the detection accuracy; however, one weakness of this approach is that it requires a certain amount of the standard templates to be provided, and once the data volume is large, the effect is not so good. Due to the continuous reduction of wafer size (Qiang et al., 2010), the effect of traditional optical detection technology is gradually getting worse.

The rapid popularity of the Convolutional Neural Network (CNN) and its excellent effects have attracted people’s attention. CNN consists of three types of layers including convolution layers, pooling layers, and fully connected layers (Saqlain et al., 2020). The convolution layer can automatically extract image features, the pooling layer can extract the main information required to create the image while reducing the number of parameters, and fully connected layers finally classify the input image using the extracted features (Krizhevsky et al., 2012). These three layers can be combined to extract the high-dimensional features of the images. In particular, the CNN models have performed well in classifying image data (Sengupta et al., 2018), and have been introduced into various industries due to their wide application. For example, to cracks in civil infrastructure (Cha et al., 2017) and classify surface defects in steel plates. The semiconductor industry has also tried to introduce CNN to improve the process for defect recognition of spring-wire sockets (Tao et al., 2018). Lee et al. (2017) designed a new CNN structure, which can identify global and invariant features in the sensor signal data, find the multivariable process fault and diagnose the fault source. Currently, deep learning methods have achieved good results in wafer detection, for example, Takeshi (Nakazawa and Kulkarni, 2018) et al. applied eight convolutional networks with activation functions to classify wafers and used simulated WMs to train a model and tested the performance on 1,191 real WMs. Cheon et al. (2019) proposed a CNN-based automatic defect classification method that can extract features from WMs and accurately classify known defect classes. The datasets used by all these studies were very small and cannot therefore fully represent the actual situation of production. CNN models can achieve higher training accuracy in the presence of bigger datasets (Najafabadi et al., 2015). Saqlain et al. (2020) proposed a deep layered CNN-based wafer defect identification (CNN-WDI) model, before training and testing the model on a real wafer dataset called WM-811K, a large dataset that consisted of eight different wafer defects and 811,457 wafer maps in total. Yu and Lu (2016) proposed a manifold learning-based wafer map defect detection and recognition system and their experimental results from WM-811K verified that the overall accuracy was 90.5%.

Noise is common in the wafer maps and can make an impact on the recognition effect, denoising can effectively preserve the defect type of the wafer and improve the accuracy. Thus, image denoising is a key step in the defect recognition procedure. Wang et al. (2006) used a spatial filter that compares the defect densities in each die of the wafer. On the other hand, noise is also a test of model robustness. When the robustness of the model is good, the impact of noise on the performance will be small. Multiscale analysis is a technique in pattern recognition and image processing that analyzes an image or pattern at various scales (Li et al., 2016a,b,c). This benefits multiple applications, such as object identification, image categorization, and feature extraction, which can help understand phenomena or processes that occur over a range of scales and for extracting features (Ataky et al., 2022). Eseholi et al. (2020) decomposed the surface profile into three multiscale filtered image types: Low-pass, Band-pass, and High-pass filtered versions, respectively, by using a Gaussian Filter. Compared to conventional roughness descriptors, their method increased surface discrimination from 65 to 81%. The term “scale” has had many meanings in metrological studies. Scale can refer to the ratio of lengths on measurement renderings to the actual lengths on the actual surface (Brown et al., 2018). In this paper, multi-scale means that the image is processed by convolution to obtain feature maps with different channel numbers. We call these feature maps with different channel numbers “multi-scale.” Through comprehensive utilization of these multi-scales, we call them “Multi-Scale Feature Fusion.” To extract patterns from observable measurements we need to be able to define and identify stable features in observable measurements (Scott, 2004), convolution can extract stable abstract features of objects, so we use a convolution neural network to extract multi-scale information.

The contributions of this paper are as follows:

1. A Multi-Feature Fusion module (MFF) is proposed based on the attributes of wafers and can combine different fine-grained features, capturing the key information from local and global regions, which can improve the robustness of wafer defect recognition.
2. A Multi-Feature Fusion Perceptual Network (MFFP-Net) is designed to integrate information from different dimensions, and the next stage can abstract features from the different scales simultaneously. Therefore, the MFFP-Net can extract more information to achieve high precision wafer recognition. It also effectively resists the interference of noise.
3. Comprehensive experiments demonstrate that the proposed method can obtain good results for identifying wafer map defect patterns, which has a recognition accuracy of 96.71% and achieves state-of-the-art wafer recognition performance in WM-811K.

## 2. Methods

In this section, we first introduce the overall structure of MFFP-Net and then introduce the composition of MFF in detail.

### 2.1. Overview

**Figure 1:**
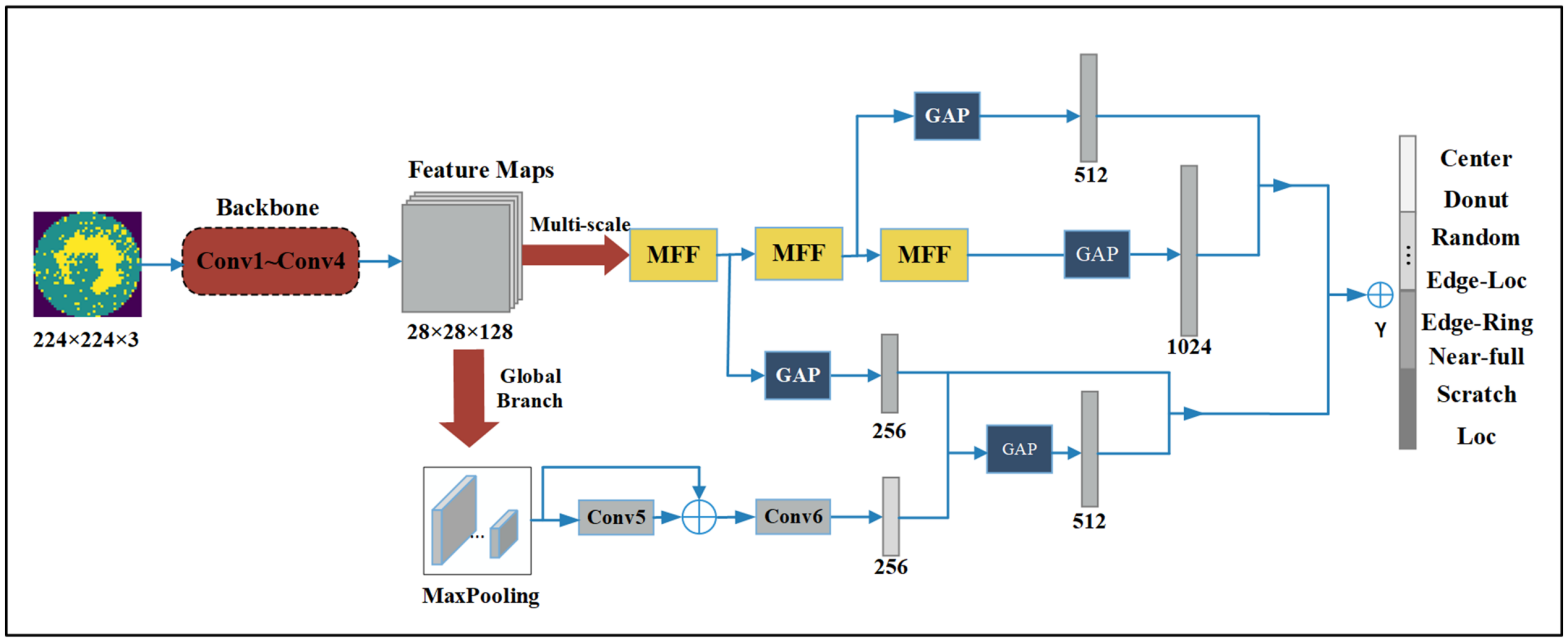
Structure of the proposed method.

We propose a Multi-Feature Fusion Perceptual Network (MFFP-Net) to address the recognition of wafer defects. As shown in Figure 1, MFFP-Net consists of four convolution layers and two branches. The network takes the original wafer defect map as input. The direction of the arrow represents the operation direction of the feature layer in turn. First, the Conv1 ~ Conv4 layer serves as the feature pre-extractor to output 28 × 28 × 128 feature maps. Then, the feature maps are input into Multi-scale Branch and Global Branch to extract different perceptual field features. The Multi-scale Branch consists of three MFF modules. The Global Branch is composed of a Max Pooling layer, Conv5, and Conv6. Finally, we fused the feature maps with 256, 512, 512, and 1,024 channels to predict the wafer defect type.

### 2.2. Backbone

**Figure 2:**
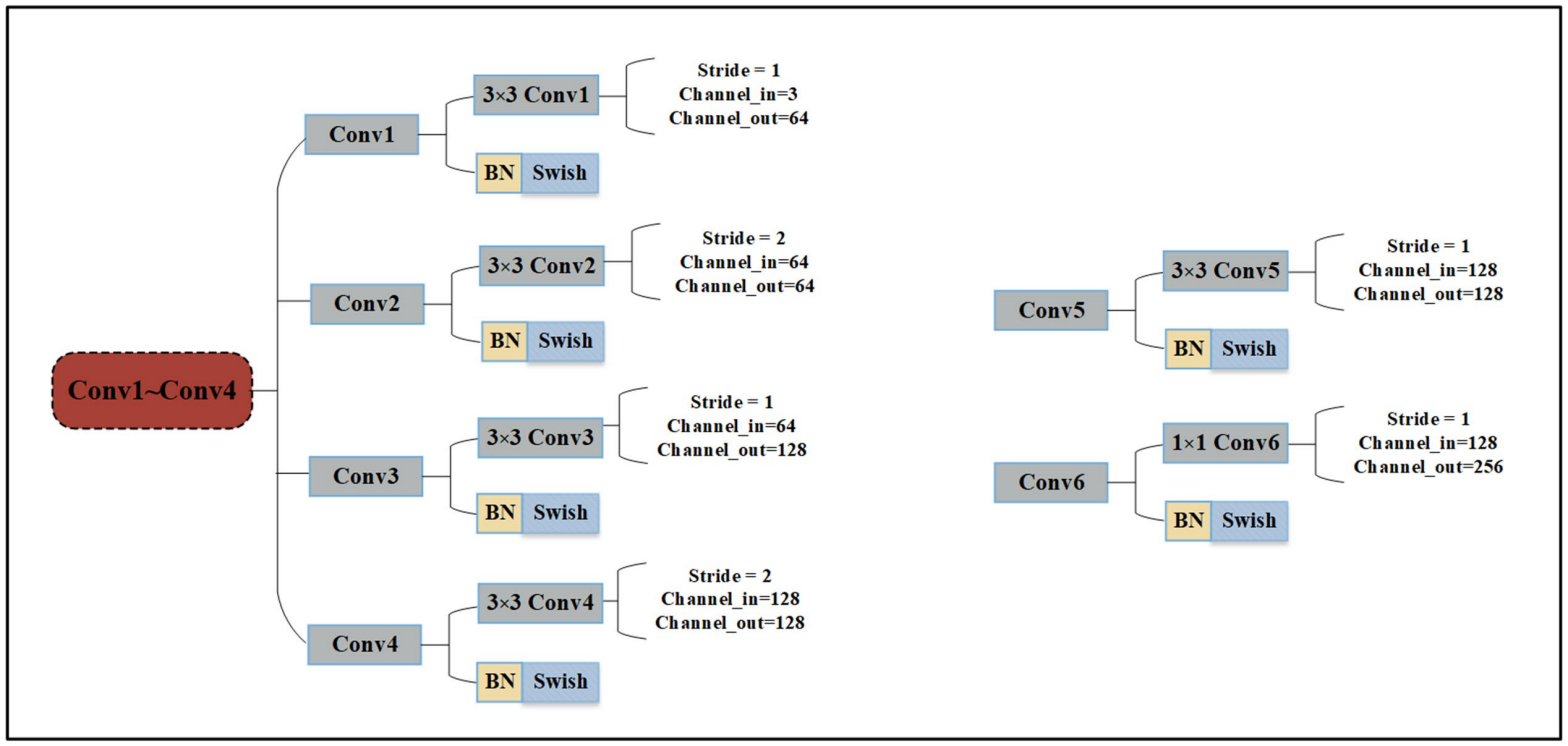
The detailed parameters of Conv1~Conv4, Conv5 and Conv6.

The Conv1 ~ Conv4 layers serve as the backbone. Then the feature maps are input into two branches to extract different perceptual field features. The Multi-scale Branch gets fine-grained features through MFFs, and the Global Branch gets features of higher dimension through further convolution operation. Finally, the recognition results are obtained by fusing the feature and decision level. GAP denotes global average pooling layers and is an element wise addition. We use the traditional convolution neural network, the most basic compositions of the neural network are convolution operation, Batch Normalization, Max pooling, and Global Average Pooling (GAP). The details of Conv1 ~ Conv4 are shown in Figure 2.

Conv1 ~ Conv4 are composed of 3 × 3 convolution operation, Batch Normalization (BN), and Swish activation function. However, the difference in this approach relates to the convolution operation parameters: including the stride operation, the input channel, and the output channel of each convolution. When the wafer image is input into the network, it will pass through Conv1 ~ Conv4 in turn. Finally, the shallow features are output by Conv4., and Conv5 and Conv6 both consist of convolution operation, BN, and Swish activation function. Conv5 uses 3 × 3 convolution and the input and output channels are 128. Conv6 uses 1 × 1 convolution to make the network deeper and the input and output channels are 128 and 256, respectively.

### 2.3. MFF module

**Figure 3:**
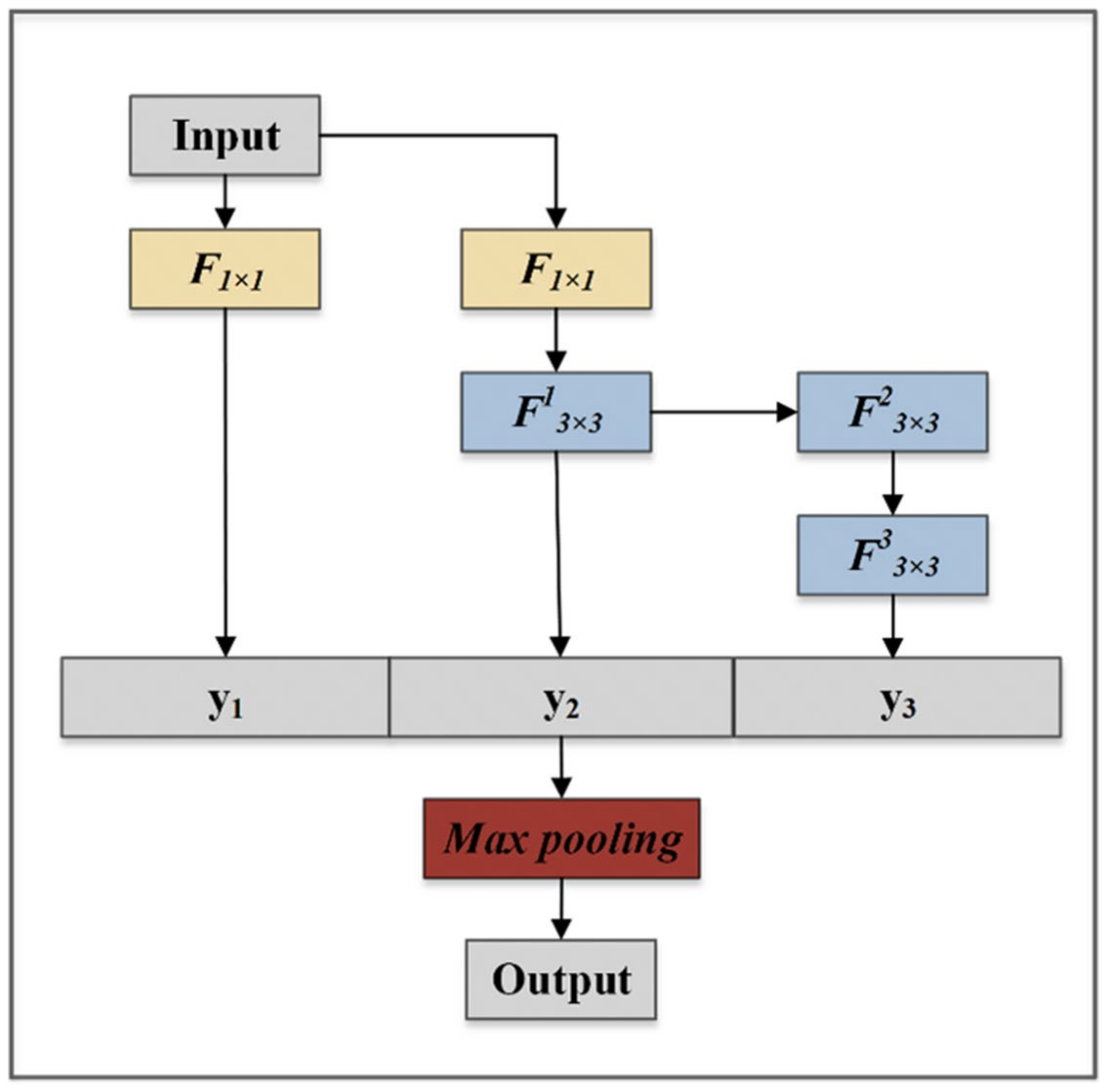
The structure of MFF module.

By controlling the longest gradient path, the deeper network can learn and converge effectively (Wang et al., 2022). The MFF module aims to obtain higher fine-grained and richer features, it uses expand and merge channels to achieve the ability to continuously enhance the learning ability of the network. As shown in Figure 3, the MFF module is composed of three branches that are composed of one, two, and four convolutions, respectively. The outputs obtained from the three branches are joined together according to dimensions. The final output is obtained after the Max Pooling layer to reduce parameters. The module follows a philosophy that visual information should be processed at various scales and then aggregated so that the next stage can abstract features from the different scales simultaneously.

The MFF module can process information at various scales and then aggregate it so that the next stage can abstract features from the different scales simultaneously. *F* denotes convolutional layers, and *y* denotes output feature maps. The arrow points to the directions in which the feature map passes.

The MFF module can be formulated by Equations (1–4).

(1) $$y_{1}=F_{1}\left(x\right)\;$$

(2) $$y_{2}=F_{3}^{1}\left(y_{1}\right)\;$$

(3) $$y_{3}=F_{3}^{3}\left[F_{3}^{2}\left(y_{2}\right)\right]\;$$

(4) $$F_{output}=MaxPool\left[Concat\left(y_{1},y_{2},y_{3}\right)\right]\;$$

Where *x* is the input of the MMF module, *F_output_* is the output of the MFF module.

*Concat* is the tensor splicing function, the dimension of the tensor can be specified for splicing.

**Figure 4:**
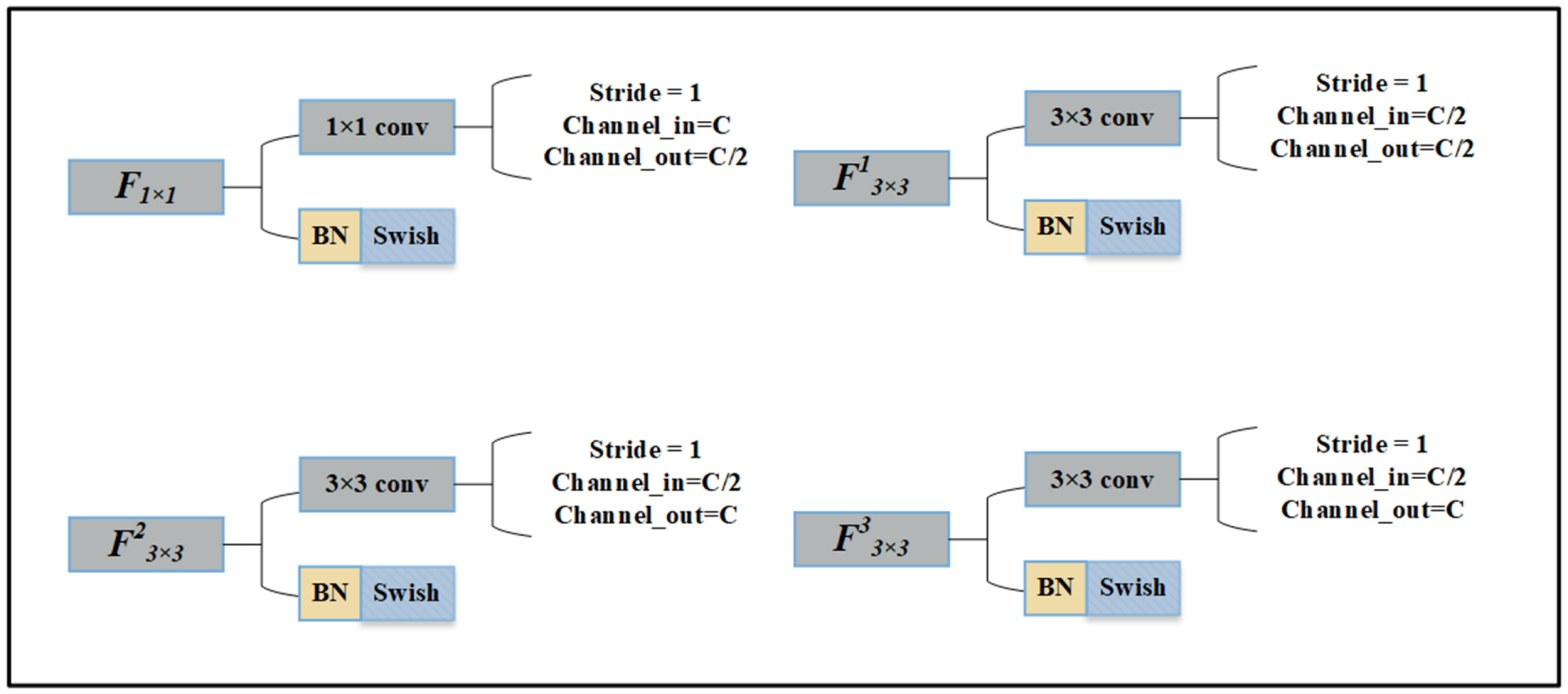
The details of 3 × 3 convolution layer and 1 × 1 convolution layer.

The details of the convolution layer are shown in Figure 4.

If the number of feature map channels input to MFF is *C*. The output channels of *F_1x1_* become *C*/2. In the convolution layer *F^1^_3x3_* next to *F_1x1_* the input and output channels are both *C*/2. The input and output channels of *F^2^_3x3_* are *C*/2 and *C*, respectively. The input and output channels of *F^3^_3x3_* are *C*. Finally, the feature map with a channel number of 2*C* is obtained.

The MMF takes the feature map obtained through the convolution layers as the input. We assume the depth (the number of channels) of the feature map is *C*.

**Figure 5:**
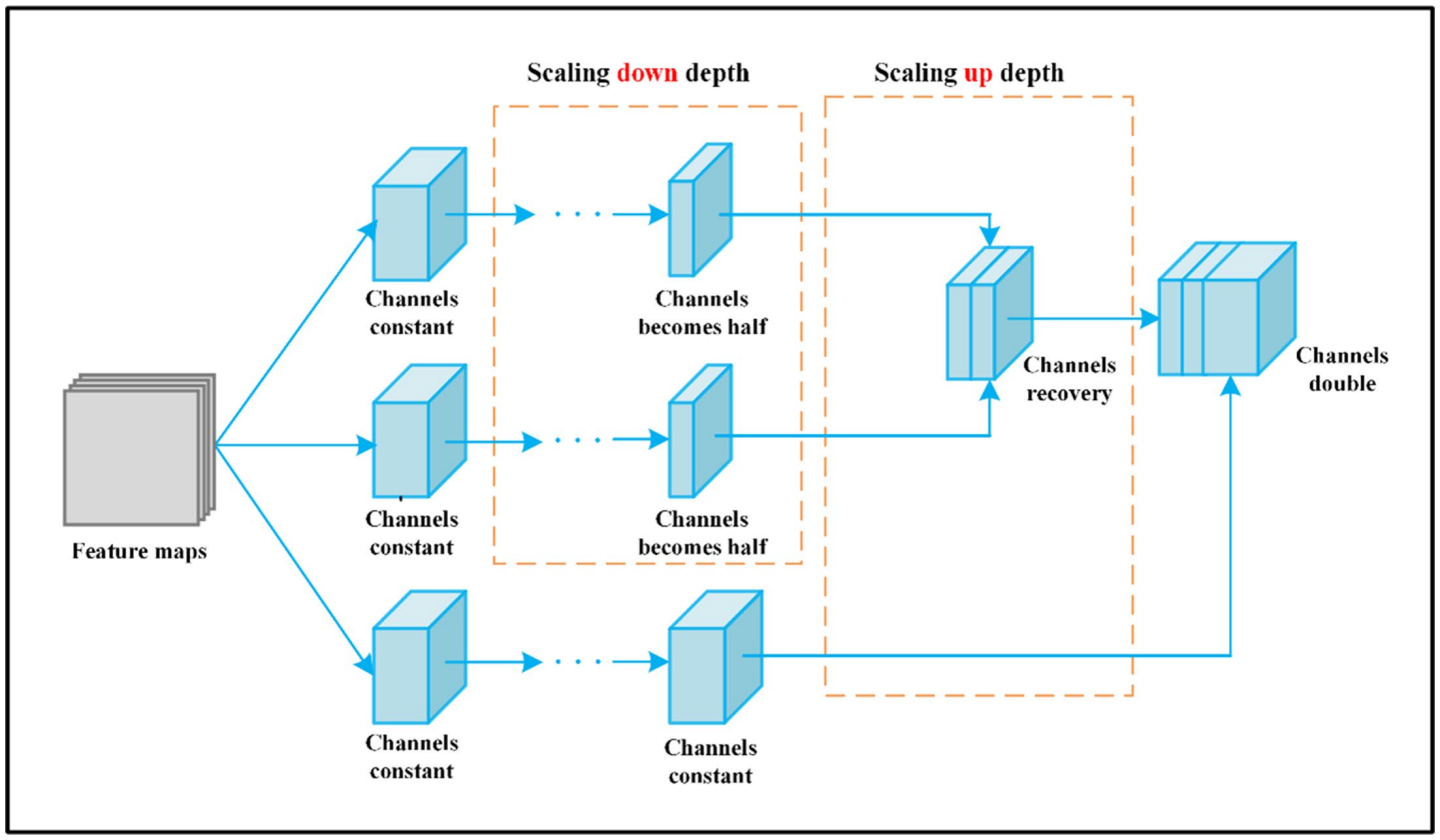
The change of channels number through MMF feature map.

The model change in depth of the feature map through MMF is shown in Figure 5.

First, a 1 × 1 convolution layer is executed after the input to adjust the number of channel dimensions and make the depth *C*/2. The introduction of 1 × 1 convolution enables the combination of channels and the interaction of information between channels. Second, after 1 × 1 convolution, the number of channels in the feature map halves, and then we use 3 × 3 convolution to further extract high-dimensional features. Third, based on the second step, after two convolution operations, the network becomes deeper and the number of channels becomes *C*. Fourth, the feature maps obtained in the first, second, and third steps are joined together according to the channel direction, the number of channels is 2*C*, and more fine-grained features are obtained. Last, feature maps with 2*C* channels pass through the convolution layer to achieve the final output.

The number of channels in the feature map is *C*. Through the first and second branches, the channels of input halves, then combine according to the channel direction, and the number of channels is still *C*. Through the third branch, the depth remains unchanged. Finally, splice feature maps with channel number *C* together and double the number of channels. *C* denotes the channels of the input. The arrow indicates the direction of channel number changes.

### 2.4. Auxiliary classifier and lead head

**Figure 6:**
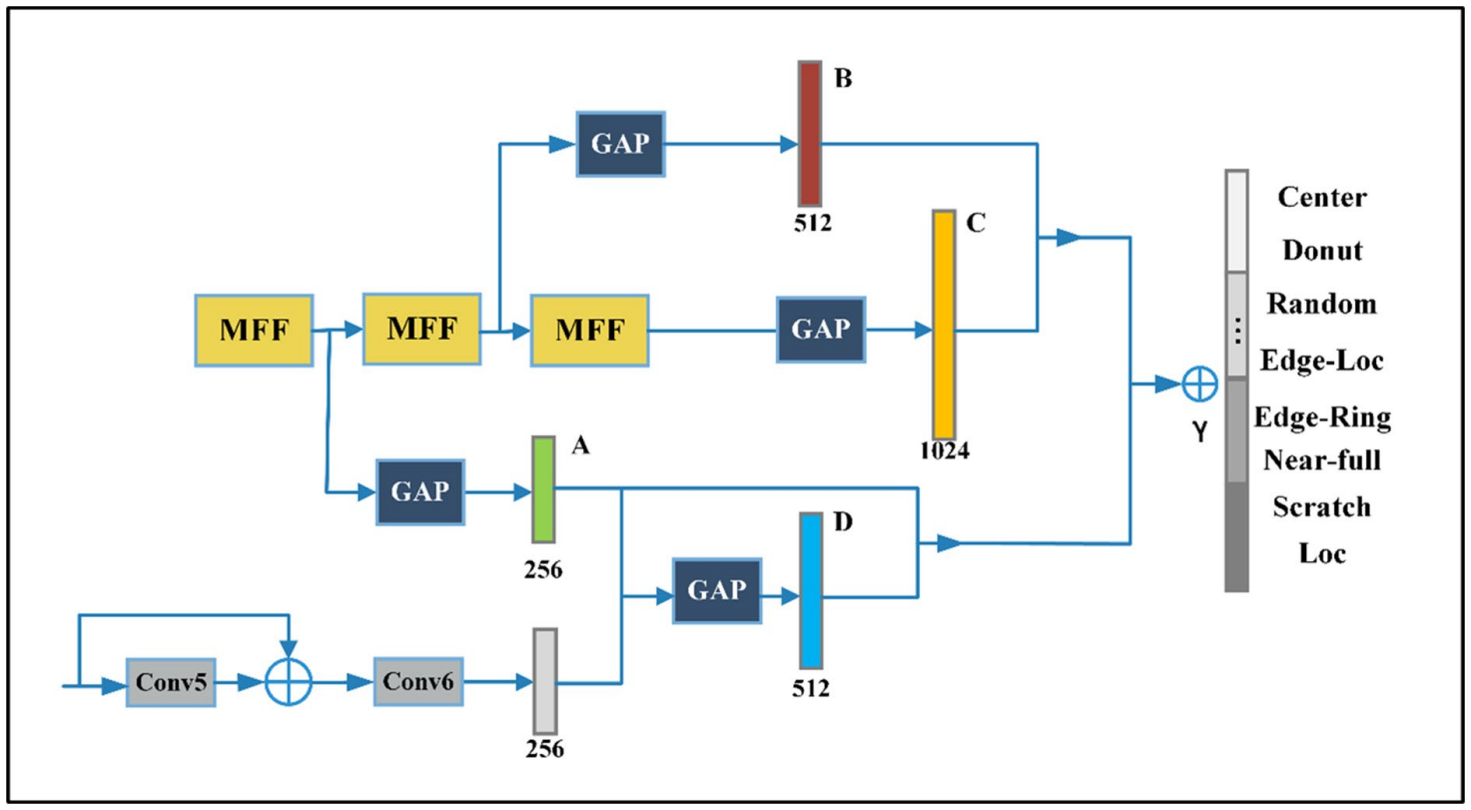
The proposed model contains four auxiliary classifiers.

Deep supervision is a technique that is often used in training deep networks. We add auxiliary head in the middle layers of the network, auxiliary head is conducted and marked as A, B, C, and D, as shown in Figure 6. The shallow network weights with assistant loss as the guide. In this paper, we refer to the classification header responsible for the final output as lead head and the head used to assist training is called auxiliary classifier. Auxiliary classifiers located at different depth levels will learn different information, and the learning ability of an auxiliary classifier is not strong as a lead head. In order to avoid losing the information that needs to learn and combine useful information together, it is crucial to find out how to assign weights to auxiliary classifiers. We will discuss the details of assigning auxiliary classifier weights in the part of Ablation Experiments. As for the output of lead head, we filter the high precision results from the high recall as the final output.

## 3. Experiments

In this section, we first introduce two datasets and their characteristics. We then explain the details of the experimental implementation. Thirdly, we adjust the parameters of the experiment to obtain the best results and visualize the effect of model recognition. Finally, we analyze the error of the experimental results.

### 3.1. Dataset

**Figure 7:**
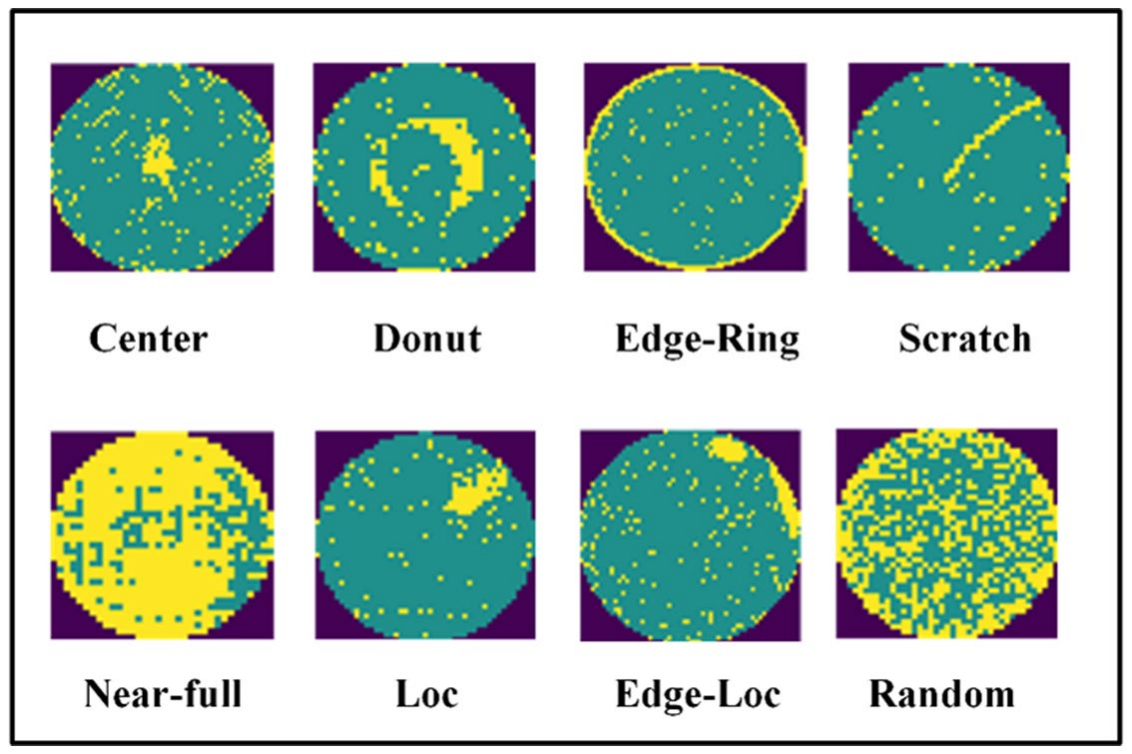
Eight different wafer defect types.

**Figure 8:**
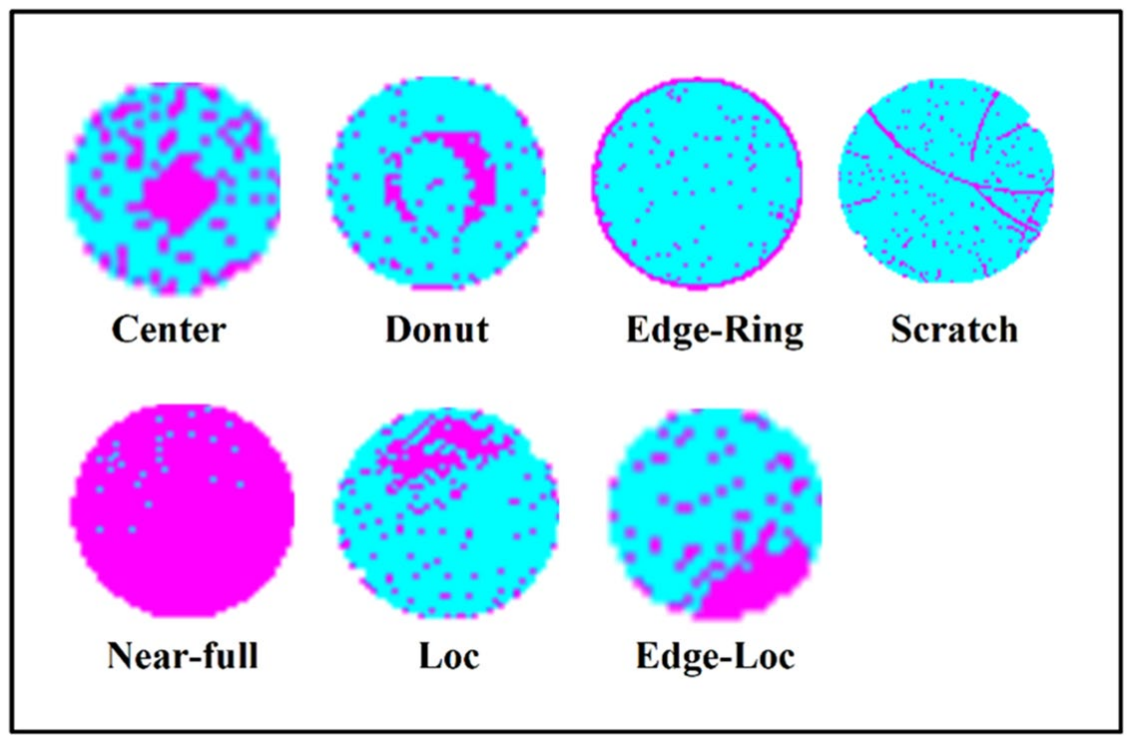
There are seven different wafer defect types in wafer-kaggle.

To compare our results with previous studies and verify the effectiveness of the method outlined in the present study, we performed experiments on real-world wafer datasets WM-811K (MIR Corpora, 2015; Wu et al., 2015). The WM-811K dataset is the largest publicly available wafer data, consisting of 811,457 wafer maps collected from 46,293 different lots in real-world fabrication. This dataset contains eight different and labeled wafer failure patterns, a total of 24,653 wafer maps, the rest were unlabeled and defect-free wafer maps. Figure 7 shows the sample wafer maps from each defect type including Center, Donut, Edge-Ring, Scratch, Near-full, Loc, Edge-Ring, and Random. The yellow part represents the defect, and the green part represents the defect-free part. Domain experts were recruited to annotate the pattern type of the wafer maps in the WM-811K dataset. We also found a data set about wafers on (Karen and Andrew, 2015) (wafer-Kaggle), shown in Figure 8.

**Figure 9:**
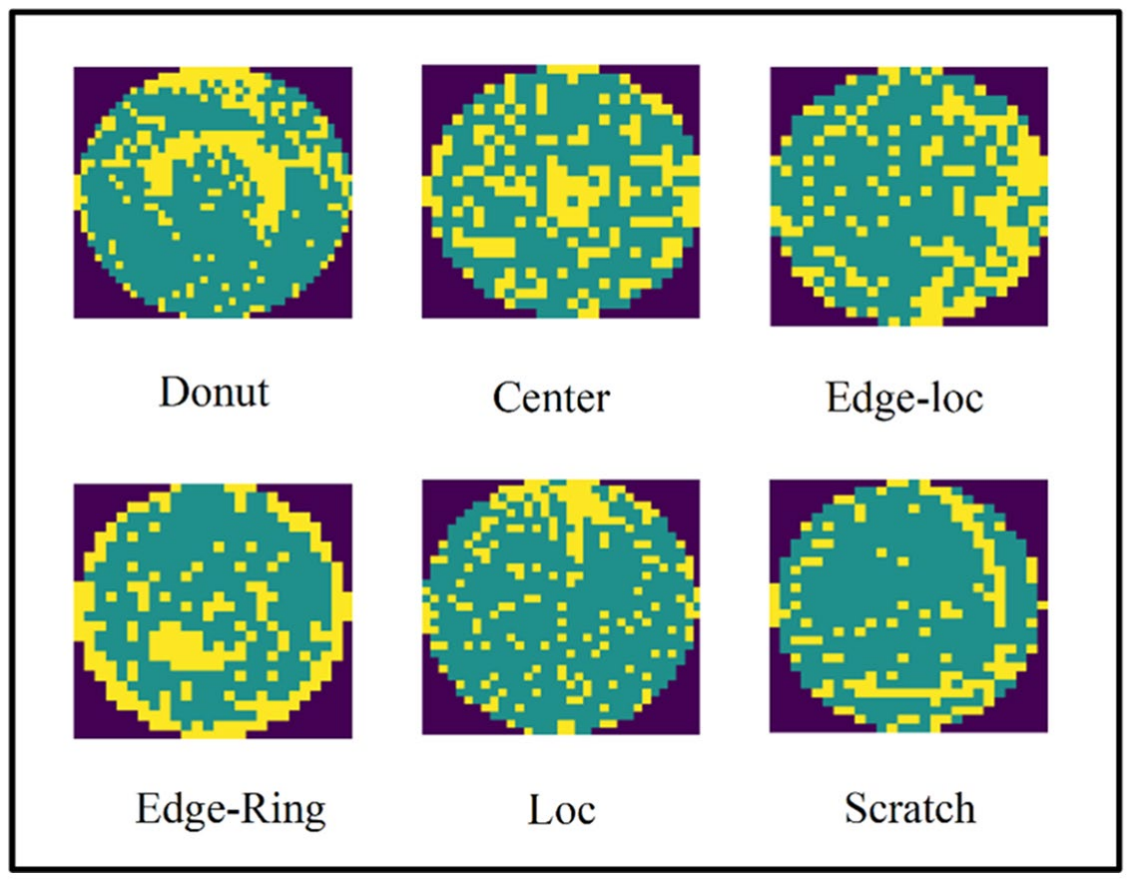
The wafer maps in WM-811K contains serious noise.

We used 25,519 wafer defect maps labeled in the WM-811K dataset to verify the performance of the model. The numbers of eight types are 4,294, 555, 5,189, 9,680, 3,593, 149, 866, and 1,193, respectively, and the proportion was 25:3:30:56:20:1:5:7. The eight wafer defect types in this data set were shown to be seriously imbalanced. The main problem in image resolution is noise and the wafer maps in the WM-811K dataset contain serious noise, as shown in Figure 9. If the robustness of the model is poor, the noise will greatly affect the performance of the model.

### 3.2. Implementation details

We divided WM-811K randomly into a training set, validation set, and test set in the ratio of 8:1:1. For the training set, we first used random clipping wafer maps, as part of which the pixel size became 224 × 224, then a random horizontal flip. For the test set, we changed the wafer map pixels to 256 × 256, then it became 224 × 224 through the center crop. The model was developed by using PyTorch. An NVIDIA 3080 GPU with 16 GB memory was engaged to accelerate the calculation. The learning rate was set to a constant of 0.0001, the weight decay coefficient was 0.05, and the minibatch size is set to 32. We train the model for a total of 100 epochs, during the training, we use Cosine Annealing with a period of 32. The number of parameters of the proposed net is 48.09 M.

### 3.3. Result and analysis

#### 3.3.1. Ablation experiments

The features produced by the layers in the middle of the network are very discriminative, even low dimensional embeddings might contain a large amount of information. To study the impact of auxiliary classifiers connected to the middle layer on classification results, the experiment with only one auxiliary classifier is conducted and marked as A, B, C, and D, as shown in Figure 6.

The impact of four different auxiliary classifiers is shown in Table 1. When there is only one auxiliary classifier, auxiliary classifier B achieved the best accuracy of 92.21%, and auxiliary classifier D achieved the lowest accuracy of 79.60%. When we use four auxiliary classifiers at the same time and give them the same weight, the accuracy is higher than when using only one auxiliary classifier, at 94.56%. Combining the features from the different scales could improve recognition accuracy.

**Table 1:** The impact of four different auxiliary classifiers on wafer classification accuracy.

(A, B)	(C, D)	Precision
(1, 0)	(0, 0)	84.58%
(0, 1)	(0, 0)	92.21%
(0, 0)	(1, 0)	88.02%
(0, 0)	(0, 1)	79.60%
(1, 1)	(1, 1)	94.56%

As shown in Table 2, when only auxiliary classifier D is used, the recognition accuracy of the model is far lower than that of other auxiliary classifiers. To study the influence of auxiliary classifier D on classification accuracy, we give different weights to D. When the weights of D are set as 1.3, 0.7, 0.5, 0.3, 0.1, and 0, respectively. The accuracy of the model is shown in Table 2, which indicates that when the A, B, C, and D ratios are 1:1:1:0.3, the model achieves the highest wafer recognition accuracy of 95.73%.

**Table 2:** The impact of auxiliary classifier D on wafer classification accuracy.

(A, B)	(C, D)	Precision (%)
(1, 1)	(1, 1.3)	87.40
(1, 1)	(1, 0.7)	88.49
(1, 1)	(1, 0.5)	94.56
(1, 1)	(1, 0.3)	95.73
(1, 1)	(1, 0.1)	95.65
(1, 1)	(1, 0)	95.07

As shown in Table 3, when only the auxiliary classifier B is used, the recognition accuracy of the model is far higher than that of other auxiliary classifiers. We fixed the weight of the auxiliary classifier D to 0.3, then set different weights for B. When the weights of B are set as 1.2, 1.4, 1.6, and 1.8 respectively, the accuracy of the model is shown in Table 3. It is indicated that when the A, B, C, and D ratios are 1:1.4:1:0.3, the model achieves the best performance.

**Table 3:** The impact of auxiliary classifier B on wafer classification accuracy.

(A, B)	(C, D)	Precision (%)
(1, 1.2)	(1, 0.3)	95.95
(1, 1.4)	(1, 0.3)	96.71
(1, 1.6)	(1, 0.3)	96.53
(1, 1.8)	(1, 0.3)	95.45

#### 3.3.2. Metrics

The methods shown in Table 4 are the results of a test run on the WM-811K dataset. As shown in Table 4, the proposed method is the best in terms of performance. The proposed method is not only simple to process but can also achieve good results.

**Table 4:** Comparison to other methods tested in the WM-811K dataset.

Model	Accuracy (%)
Ours	96.71
CNN-WDI (Saqlain et al., 2020)	96.20
SVE (Saqlain et al., 2019)	95.86
YOLOV4 (Shinde et al., 2022)	95.70
WMFPR (Wu et al., 2015)	94.63
YOLOV3 (Shinde et al., 2022)	94.40
CVAE (Ho et al., 2021)	93.60
SCSDAE (Yu et al., 2019)	92.63
Label reconstruction (Park and Jang, 2021)	91.20
DTE-FPR (Piao et al., 2018)	90.50

We used the same settings as the proposed model to test some common classified networks. As shown in Table 5, our model is 1.48% higher than that ranked second place, ResNet50.

**Table 5:** Comparison to other models tested in the WM-811K dataset.

Model	Accuracy (%)
Ours	96.71
ResNet50 (He et al., 2015)	95.23
VGG16 (Karen and Andrew, 2015)	95.20
MobileNet (Andrew et al., 2017)	93.20
GoogleNet (Christian et al., 2015)	93.82
ResNet34 (He et al., 2015)	92.64
ResNet101 (He et al., 2015)	91.04

We also used the True Positive Rate (TPR) and True Negative Rate (TNR) as metrics to measure the performance of the model. TPR is the proportion of positive examples predicted by the model to all real positive examples. TNR is the proportion of negative examples predicted by the model to all real negative examples. TPR and TNR are calculated by Equations (5, 6), respectively.

(5) $$TPR=\frac{TP}{TP+FN}\;$$

(6) $$TNR=\frac{TN}{TN+FP}\;$$

TP is the number of positive examples correctly classified by the model.

FN is the number of positive examples incorrectly classified by the model.

FP is the number of negative examples incorrectly classified by the model.

TN is the number of negative examples correctly classified by the model.

As shown in Table 6, model performance in WM-811K, for other types except for Near-full, the recognition precision is above 88%. For Random and Edge-Ring, the precision is more than 99%. The reason for the low recognition accuracy of Near-full will be discussed in the error analysis. The specificity for all kinds of wafers exceeds 99%.

**Table 6:** The performance of the proposed model in WM-811K.

Defect type	Precision (%)	TPR (%)	TNR (%)
Loc	95.3	89.4	99.3
Center	96.8	99.5	99.3
Donut	88.5	96.7	99.7
Random	100.0	88.5	100.0
Scratch	95.6	90.0	99.8
Near-full	75.0	100.0	99.8
Edge-loc	94.8	97.5	98.6
Edge-Ring	99.1	99.3	99.4

We also tested the proposed model in wafer-Kaggle, as shown in Table 7, the recognition accuracy of each type of wafer was more than 87%. The recall was more than 87% and the specificity exceeded 98%. The precision of Near-full was 100%.

**Table 7:** The performance of the proposed model in wafer-Kaggle.

Defect type	Precision (%)	Recall (%)	Specificity (%)
Loc	88.5	85.8	98.8
Center	97.2	99.7	99.3
Donut	93.0	97.6	99.8
Scratch	87.5	98.0	99.6
Near-full	100.0	100.0	100.0
Edge-loc	93.0	87.2	98.9
Edge-Ring	99.1	99.4	99.1

**Figure 10:**
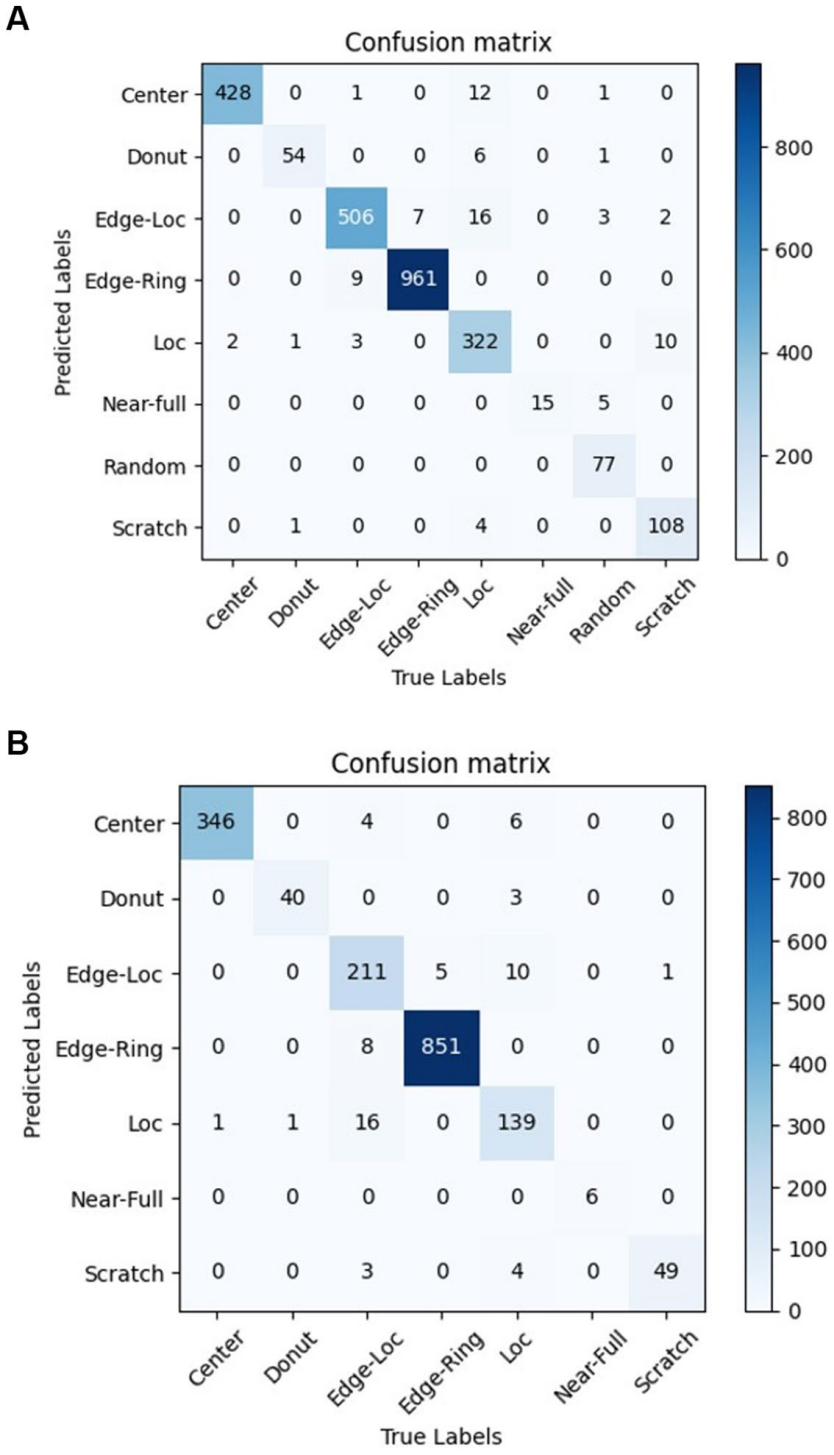
Confusion matrix of the WM-811K and wafer-Kaggle datatset. **(A)** Confusion matrix of the WM-811K. **(B)** Confusion matrix of the wafer-Kaggle datatset.

The confusion matrix of the WM-811K and wafer-Kaggle dataset are shown in Figure 10.

#### 3.3.3. Visualization

**Figure 11:**
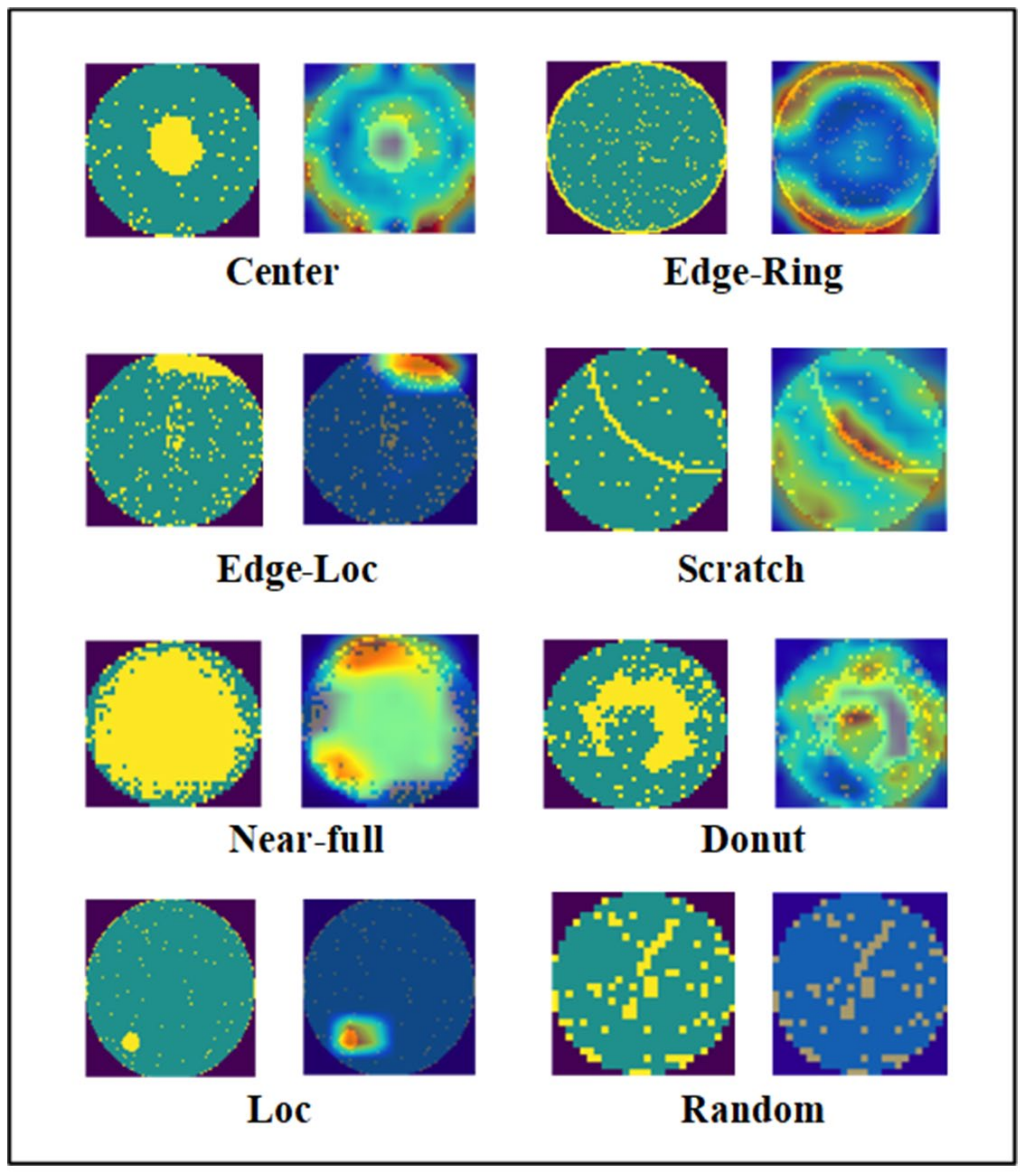
Attention maps of eight different wafers in WM-811K.

To further investigate the performance of the proposed model in more detail, we use gradient weighted class activation mapping (Grad-CAM) (Du and Martinez, 2011) to visualize it. As shown in Figure 11, when the area more brightly colored, the model pays more attention to it. For different types of wafer defects, the proposed model can capture their unique features accurately and not be affected by noise.

**Figure 12:**
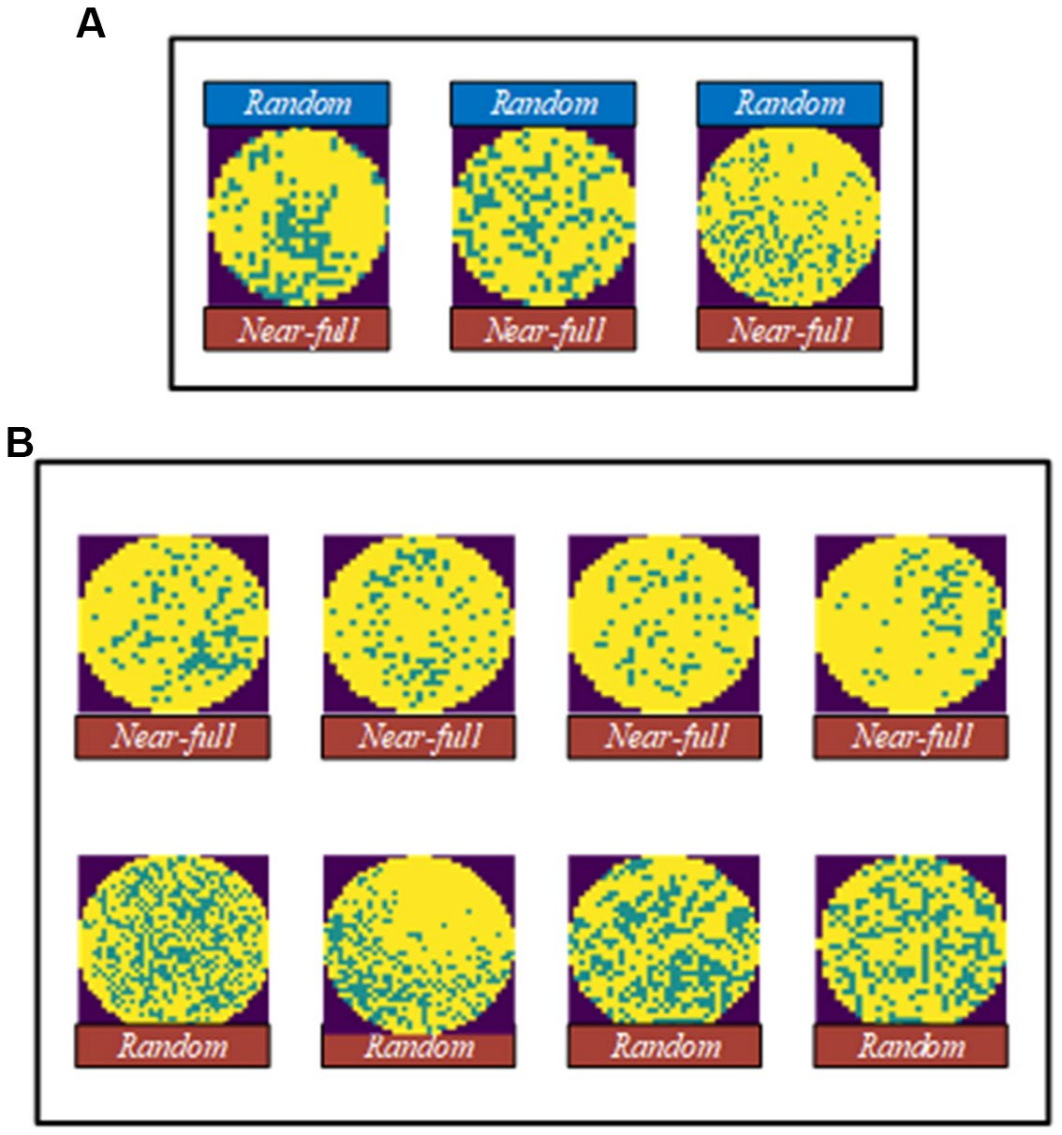
Error analysis. **(A)** Water images with random defect in the WM-811K dataset where MFFP-Net failed to predict the correct defect categories. **(B)** Comparison between near-full and random.

Figure 12 shows wafer images with Random defects in the WM-811K dataset where MFFP-Net failed to predict the correct defect categories. Although MFFP-Net is robust to wafer maps with noise, great similarity between Random and Near-full leads to recognition errors. The solution to the problem is to supplement more information about these two defect types, such as using multiple data enhancement methods to increase differences.

## 4. Conclusion and discussion

This paper proposes a Multi-Feature Fusion Perceptual Network (MFFP-Net) inspired by the attributes of the wafers and human visual perception mechanism to recognize wafer defects. We designed a multi-feature fusion module through which information can be processed at various scales and then aggregated so that the next stage can abstract features from the different scales simultaneously. The final experiment and comparison with existing methods showed that the proposed method can effectively eliminate the influence of noise and achieve high precision recognition. DNA computing is a novel intelligent method that can be applied to remote sensing image classification (Jiao et al., 2010) and sodar data classification (Ray and Mondal, 2011). Due to DNA computing having the characteristics of massive parallel computing, in future work, we plan to explore using it to classify wafers and compared it with the method based on neural networks in performance.

## Data availability statement

The original contributions presented in the study are included in the article/supplementary material, further inquiries can be directed to the corresponding author.

## Author contributions

YC: data curation and writing—original draft. ZX: formal analysis. MZ and JJ: project administration. KL and JJ: writing—review and editing. All authors have read and agreed to the published version of the manuscript.

## Funding

This work was supported by the Natural Science Foundation of Shaanxi Province (2023-JC-YB-49).

## Conflict of interest

The authors declare that the research was conducted in the absence of any commercial or financial relationships that could be construed as a potential conflict of interest.

## Publisher’s note

All claims expressed in this article are solely those of the authors and do not necessarily represent those of their affiliated organizations, or those of the publisher, the editors and the reviewers. Any product that may be evaluated in this article, or claim that may be made by its manufacturer, is not guaranteed or endorsed by the publisher.
